# Return to different climate states by reducing sulphate aerosols under future CO_2_ concentrations

**DOI:** 10.1038/s41598-020-78805-1

**Published:** 2020-12-10

**Authors:** Toshihiko Takemura

**Affiliations:** Research Institute for Applied Mechanics, Kyushu University, Fukuoka, Japan

**Keywords:** Climate and Earth system modelling, Atmospheric science

## Abstract

It is generally believed that anthropogenic aerosols cool the atmosphere; therefore, they offset the global warming resulting from greenhouse gases to some extent. Reduction in sulphate, a primary anthropogenic aerosol, is necessary for mitigating air pollution, which causes atmospheric warming. Here, the changes in the surface air temperature under various anthropogenic emission amounts of sulphur dioxide (SO_2_), which is a precursor of sulphate aerosol, are simulated under both present and doubled carbon dioxide (CO_2_) concentrations with a climate model. No previous studies have conducted explicit experiments to estimate the temperature changes due to individual short-lived climate forcers (SLCFs) in different climate states with atmosphere–ocean coupled models. The simulation results clearly show that reducing SO_2_ emissions at high CO_2_ concentrations will significantly enhance atmospheric warming in comparison with that under the present CO_2_ concentration. In the high latitudes of the Northern Hemisphere, the temperature change that will occur when fuel SO_2_ emissions reach zero under a doubled CO_2_ concentration will be approximately 1.0 °C, while this value will be approximately 0.5 °C under the present state. This considerable difference can affect the discussion of the 1.5 °C/2 °C target in the Paris Agreement.

## Introduction

Sulphate has natural origins and is also a primary anthropogenic aerosol in the atmosphere. The main precursor gases of sulphate aerosols are sulphur dioxide (SO_2_), of which volcanoes and fossil fuels are the major natural and anthropogenic sources, respectively, and dimethyl sulphide (DMS), which is emitted from oceanic phytoplankton and land vegetation as natural sources. SO_2_ is also emitted by biomass burning, which is currently dominated by anthropogenic causes. Sulphate aerosols alter the atmospheric solar radiation budget, consequently affecting climate change through aerosol–radiation interactions by scattering radiation and through aerosol–cloud interactions by changing the microphysical properties of water clouds by acting as cloud condensation nuclei. In recent decades, several studies have estimated the effects of anthropogenic sulphate aerosols on radiative forcing, i.e., changes in the radiation budget. The latest Assessment Report of the Intergovernmental Panel on Climate Change (IPCC AR5)^1^ evaluated the instantaneous radiative forcing of anthropogenic sulphate aerosols due to aerosol–radiation interactions to be − 0.40 W m^–2^, with uncertainty levels of − 0.20 to − 0.60 W m^–2^ in the global and annual means. The global and annual mean radiative forcing of total anthropogenic aerosols due to aerosol–cloud interactions was estimated as − 0.45 W m^–2^ by IPCC AR5^1^ with great uncertainty from 0 to − 1.2 W m^–2^. Aerosol–cloud interactions include changes in radiation scattering and absorption via changes in cloud particle size, as well as meteorological field perturbations, i.e., changes in the amount of clouds; thus, the values estimated by the IPCC AR5^1^ represent effective radiative forcing.

Although IPCC AR5^1^ did not independently estimate radiative forcing by aerosol–cloud interactions caused by anthropogenic sulphate aerosols, the total radiative forcing by sulphate aerosols is clearly a primary component of anthropogenic negative radiative forcing. Therefore, anthropogenic sulphate aerosols are expected to mitigate global warming by greenhouse gases to some extent. Simulations of the effects of sulphate aerosols on past, present, and future climate change by general circulation models were included in contributions by the Coupled Model Intercomparison Projects (CMIPs) to the IPCC Assessment Reports and recent studies^2,3^, in which climate change (e.g., changes in temperature and precipitation) associated with the overall reduction of anthropogenic aerosol-related components was assessed. However, the contribution of each component among short-lived climate forcers (SLCFs), including aerosols, to changes in surface air temperature is poorly understood, despite the estimation of instantaneous and effective radiative forcing by each component in past studies and in IPCC AR5^1^. Recent studies have indicated that the spatial pattern of the temperature response is not very consistent with that of the radiative forcing with changing in sulphate aerosol concentration^4^. The climate sensitivity of the changes in the surface air temperature per unit of instantaneous radiative forcing differs greatly among components^5–7^. This finding indicates that radiative forcing alone is not an appropriate index to quantitatively estimate climate change due to external climate forcing agents, and therefore, it is essential to use simulations from coupled atmosphere–ocean general circulation models.

The climate sensitivities of particular climate forcing agents may also depend on the climate state, e.g., mean temperature level. In particular, the sensitivities of SLCFs under different climate states should be quantitatively understood because some of the anthropogenic emissions related to SLCFs are being reduced due to measures against air pollution; on the other hand, atmospheric CO_2_ concentrations are expected to continue to increase for at least next several decades. In this study, unprecedented sensitivity experiments of surface air temperature to changing sulphate aerosol concentration, which is a primary anthropogenic aerosol, and at CO_2_ concentrations higher than the current level are performed with a coupled atmosphere–ocean general circulation model.

## Results and discussion

This study used a general circulation model coupled with an aerosol process model, MIROC-SPRINTARS^8–10^, which calculates the global spatiotemporal distributions of the mass mixing ratios of each aerosol component as prognostic variables. The model incorporates changes in the meteorological field through radiation and cloud precipitation processes as aerosol–radiation and aerosol–cloud interactions, respectively, using the predicted aerosol mass mixing ratio. The simulated results by MIROC-SPRINTARS have been confirmed to be appropriate by various methods, including comparisons among models and with observations, e.g., Aerosol Comparisons between Observations and Models (AeroCom)^11–13^. Sensitivity experiments were performed using perturbed SO_2_ emissions from a realistic range of fuel sources (factors of 0.0, 0.1, 0.3, 0.5, 0.8, 1.5, and 2.0 relative to present emissions) under present (369 ppm, recorded in 2000) and doubled (738 ppm, close to the SSP3-7.0 scenario for 2080) CO_2_ concentration levels. The SSP3-7.0 scenario is published as a Shared Socioeconomic Pathways (SSP)^14,15^ future scenario used in the IPCC 6th Assessment Report (AR6) in 2021; the scenario is used as the standard future scenario in the Aerosol Chemistry Model Intercomparison Project (AerChemMIP)^16^, which will contribute to IPCC AR6. In SSP3-7.0, greenhouse gas emissions are close to the total Nationally Determined Contributions submitted by each country to the United Nations Framework Convention on Climate Change (UNFCCC) for 2030 and are projected to continue to increase at the same rate until late in the twenty-first century. The model and experimental settings are described in detail in the “Methods” section. The single-model approach does not include differences in physical representation, which enables consistent sensitivity experiments.

**Figure 1:**
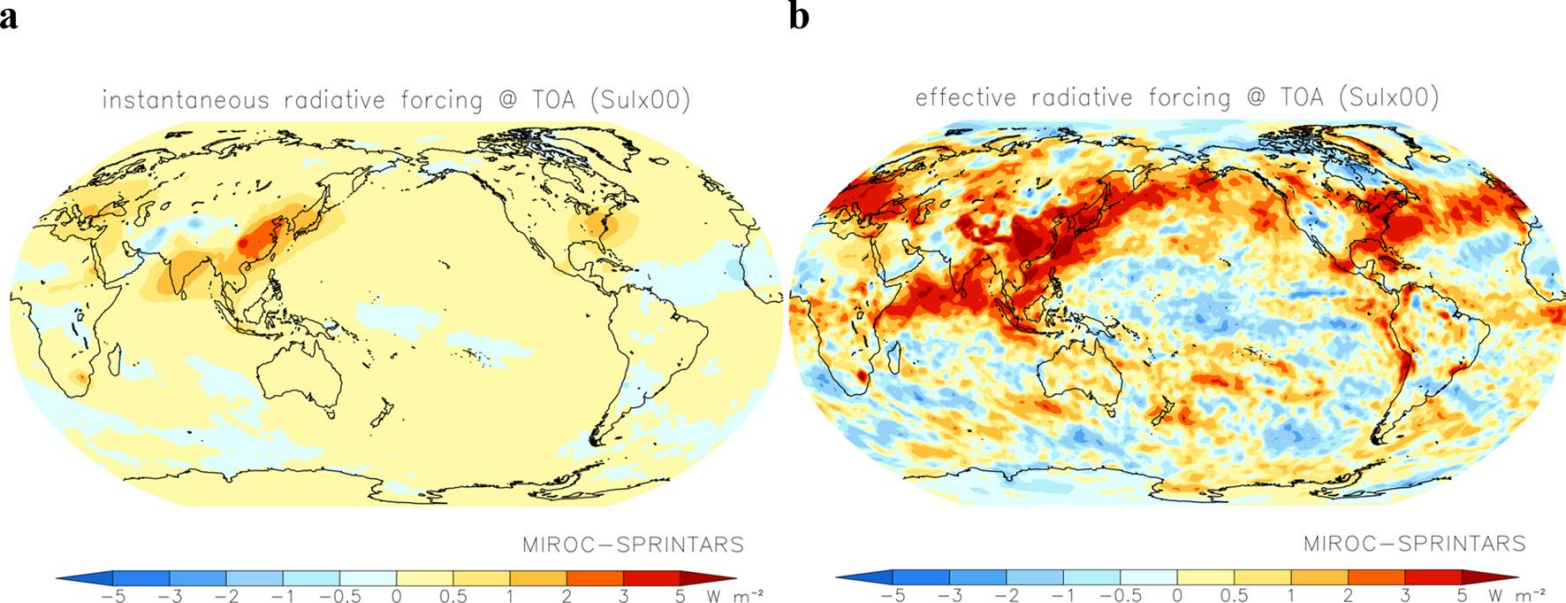
Global distribution of radiative forcing with reduced sulphate aerosols. Distribution of the annual mean instantaneous (**a**) and effective (**b**) radiative forcing if fuel SO_2_ emissions are reduced to zero simulated by the MIROC–SPRINTARS. The maps were generated with GrADS 2.2.1 (http://cola.gmu.edu/grads/).

Figure 1 shows the annual mean instantaneous and effective radiative forcing if fuel SO_2_ emissions are reduced to zero with the present CO_2_ concentration simulated by MIROC-SPRINTARS. The instantaneous radiative forcing was attributed to aerosol–radiation interactions and roughly represent a change in sulfate aerosol concentration. It was largest over East and South Asia, followed by the concentrations in Southeast Asia, North and Central America, and Europe. The large effective radiative forcing was extended to oceanic outflow regions of sulphate aerosols, especially over tropical Asia and storm tracks in the North Pacific and North Atlantic due to aerosol–cloud interactions where the cloud water content is high.

**Figure 2:**
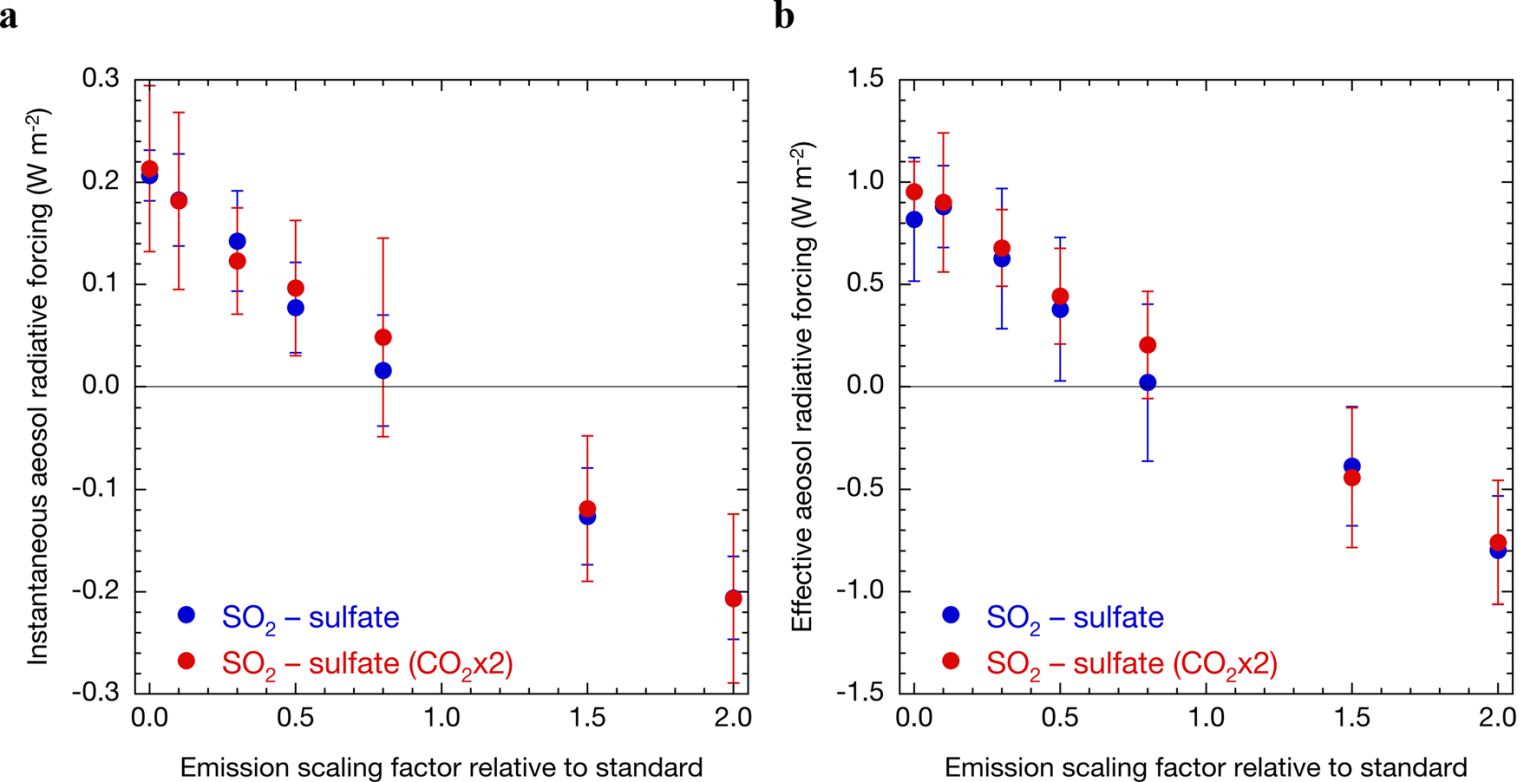
Sensitivity of radiative forcing to changes in SO_2_ emissions. Global and annual mean instantaneous aerosol radiative forcing (**a**) and effective radiative forcing (**b**) under present (blue) and doubled (red) CO_2_ concentrations with the SO_2_ emission perturbations due to fuel sources relative to present emissions simulated by the MIROC–SPRINTARS. Error bars represent the standard deviation of the global and annual means obtained from the analyzed 10-year integrals.

Instantaneous radiative forcing of sulphate aerosols shows a linear trend with perturbed SO_2_ emissions under both present and doubled CO_2_ concentrations (Fig. 2a). There was no consistent quantitative trend showing the dependence of radiative forcing on the CO_2_ levels. Although effective radiative forcing is larger than instantaneous radiative forcing due to the inclusion of aerosol–cloud interactions, it showed linear trends with changing SO_2_ emissions, which was also not dependent on the CO_2_ concentration (Fig. 2b). The radiative forcing values simulated by MIROC-SPRINTARS were within the ranges estimated by IPCC AR5^1^.

**Figure 3:**
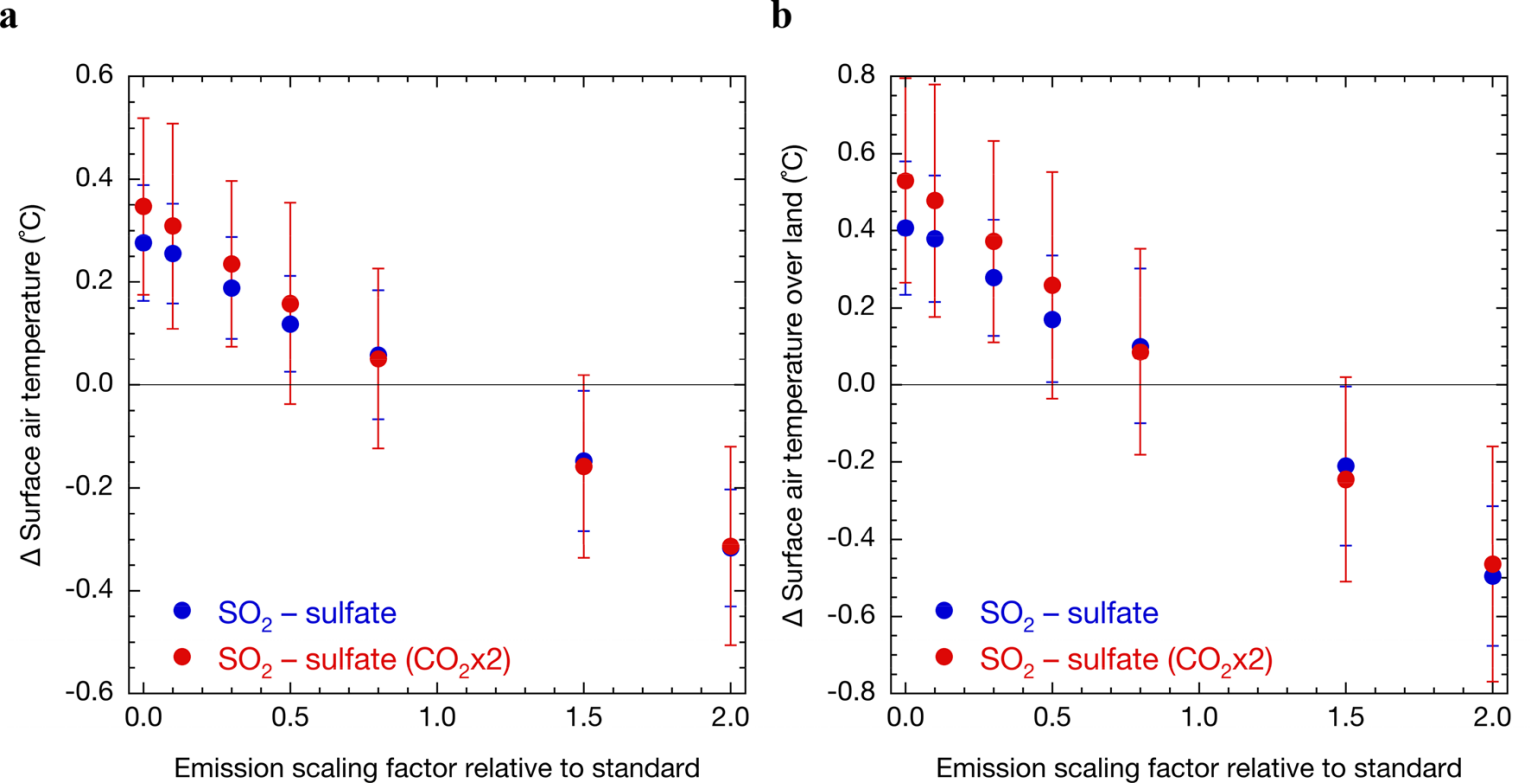
Sensitivity of surface air temperature to changes in SO_2_ emissions. The global average (**a**) and land average (**b**) changes of the annual mean surface air temperature under present (blue) and doubled (red) CO_2_ concentrations with the SO_2_ emission perturbations due to fuel sources relative to present emissions simulated by the coupled atmosphere–ocean MIROC-SPRINTARS. Error bars represent the standard deviation of the global and annual means obtained from the analyzed 50-year integrals.

Although changes in surface air temperature due to the concentration or perturbations of each SLCF have not been assessed, even in the latest IPCC Assessment Report^1^, we implicitly estimated these changes from radiative forcing since changes in the radiation budget are a primary climate change index. However, as explained above, recent studies have shown that changes in surface air temperature differ greatly between sulphate and black carbon (BC) aerosols^5–7^, even at the same level of radiative forcing. From a different perspective, Fig. 3 shows the dependency of surface air temperature on the CO_2_ concentration due to sulphate aerosols, despite a lack of dependence of its radiative forcing on CO_2_ concentration (Fig. 2). The changes in the global mean surface air temperature with zero anthropogenic SO_2_ emissions at the present and doubled CO_2_ concentrations were 0.28 and 0.35 °C, respectively, and the difference was statistically significant with 99% confidence intervals (Fig. 3a). The difference was also statistically significant with 95% confidence intervals for 0.1 and 0.3 times anthropogenic SO_2_ emissions. Interannual variability of the change in the surface air temperature with the SO_2_ emission perturbations is larger in the higher CO_2_ concentration. The mean temperature increase over land was enhanced by approximately one-third under doubled CO_2_ concentrations when SO_2_ emissions were largely reduced (Fig. 3b). While there was no significant difference in the effective radiative forcing (Fig. 2b), which is an index of rapid adjustment in the atmosphere, the differences in the surface air temperature increase at large reduction of anthropogenic SO_2_ emissions depending on the CO_2_ concentration were significant because the radiative imbalance due to sulphate aerosols results in mainly a slow climate response^17^.

**Figure 4:**
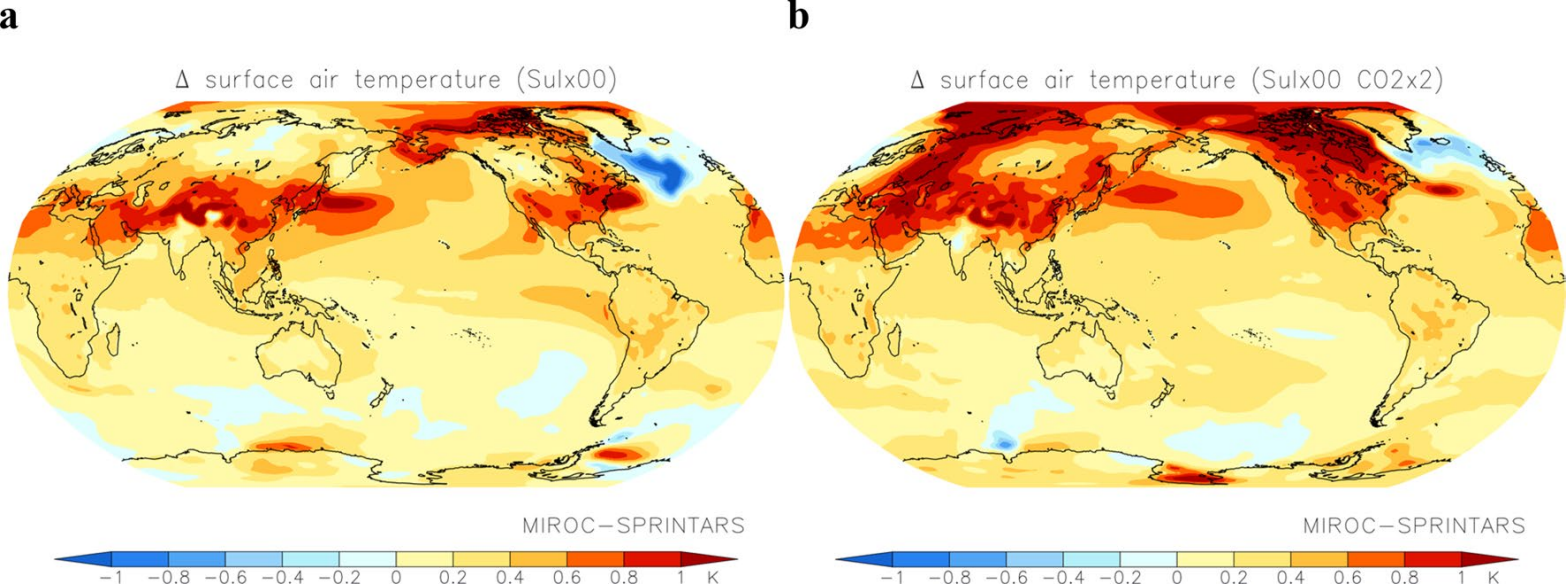
Changes in surface air temperature due to reducing SO_2_ emissions to zero. Annual mean distribution of changes in surface air temperature following reduction of SO_2_ emissions from fuel sources to zero under present (**a**) and doubled (**b**) CO_2_ concentrations simulated by the coupled atmosphere–ocean MIROC-SPRINTARS. The maps were generated with GrADS 2.2.1 (http://cola.gmu.edu/grads/).

**Figure 5:**
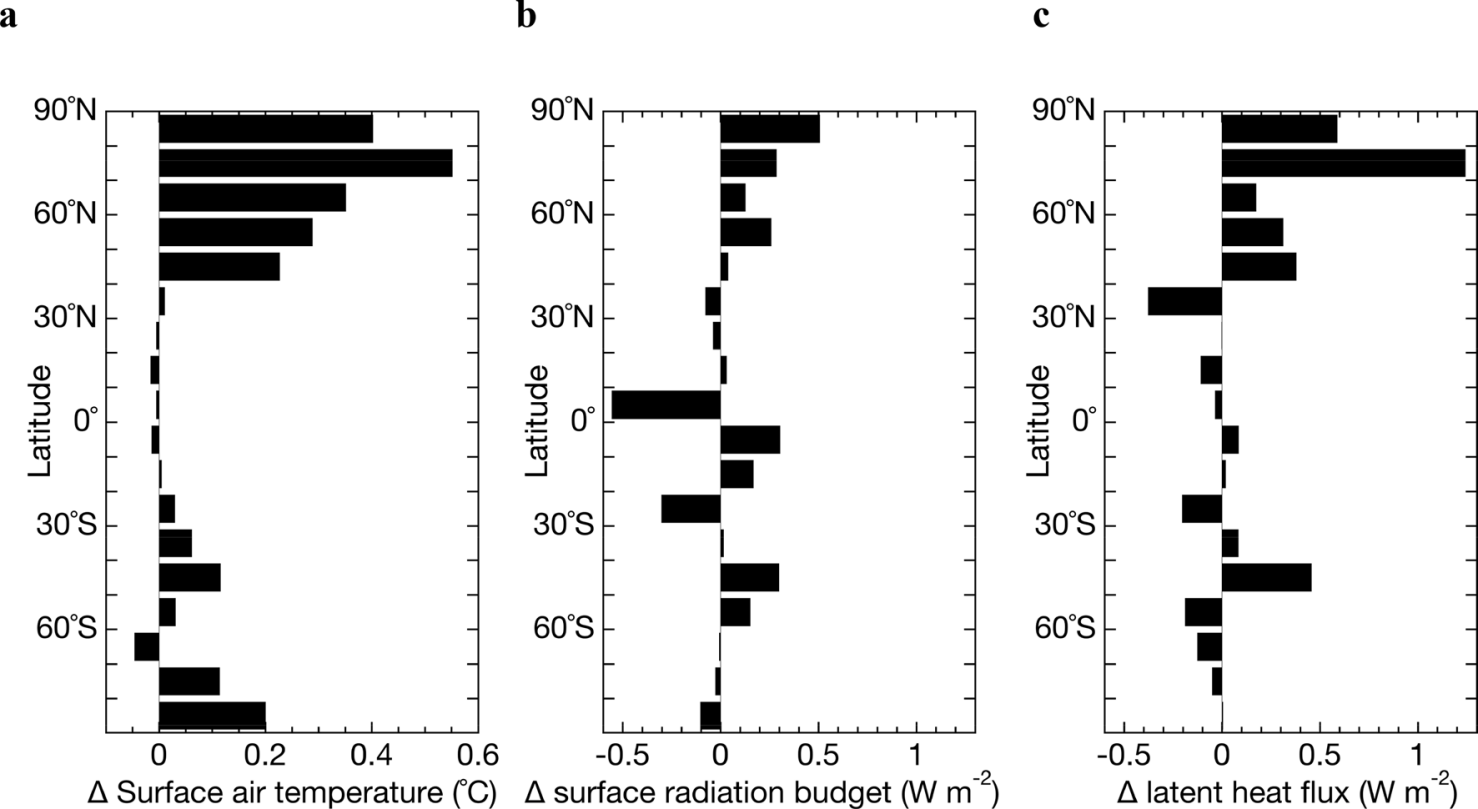
Factors of the temperature difference depending on CO_2_ concentrations. The zonal averaged differences under doubled CO_2_ concentration relative to the present in changes in the surface air temperature (**a**), shortwave plus longwave radiation budget at the surface (downward positive) (**b**), and latent heat flux (**c**) following reduction of SO_2_ emissions from fuel sources to zero.

As shown in Fig. 4, warming due to the reduction in sulphate concentrations was strong over land in the mid-latitudes of the Northern Hemisphere and the northwestern Pacific, which is a region that experiences outflow air pollutants from East Asia. The geographical pattern under the present CO_2_ concentration (Fig. 4a) was similar to that of the effective radiative forcing (Fig. 1b), although there is a change similar to the “warming hole” in the North Atlantic which is indicated to occur as global warming progresses^18^. Temperature increases of 0.5 to > 1.0 °C were projected over these regions when SO_2_ emissions from fuel sources reached zero, indicating that SO_2_ emissions cannot be neglected in comparisons of global warming due to greenhouse gases. Under doubled CO_2_ concentration scenarios, strong warming was also expected over land in the high latitudes of the Northern Hemisphere and Arctic Ocean (Fig. 4b). The differences in the zonal and annual mean temperature changes with reaching zero fuel SO_2_ emissions under present (approx. 0.5 °C) and doubled CO_2_ concentrations (approx. 1.0 °C) range from 0.4 to 0.6 °C (Fig. 5a). This result suggests that the climate response due to increased incident solar radiation at the surface with reduced sulphate aerosols will be more sensitive under warmer climates.

A large zonal mean difference was found in the change in the surface radiation budget north of 50°N following reduction of fuel SO_2_ emissions to zero with doubled CO_2_ concentration relative to the present, ranging from 0.2 to 0.5 W m^–2^ (Fig. 5b). Most of this difference is due to a change in shortwave radiation, and then it can be attributed to the magnitude of the ice–albedo feedback, which is due to the warming associated with reduced SO_2_ emissions. A change in the surface albedo at the shortwave radiation was larger by about 5 to 10% or more in the Arctic Ocean and Hudson Bay and by a few percent over the North America under doubled CO_2_ concentration (Fig. S1a). This is consistent with a change in the sea ice extent (Fig. S1b). Even for the same amount of SO_2_ emission reduction, the higher the CO_2_ concentration, the greater the ice albedo feedback. The large temperature changes in the Arctic during the northern hemisphere autumn when the amounts of sea ice and surface snow are at their minimum even at current levels of CO_2_ concentration also indicate that the ice–albedo feedback is one of the major factors (Fig. S2).

A difference in the latent heat change is also considered to be an important factor in the difference in sensitivity to temperature changes due to sulfate aerosols. The difference in latent heat change was large north of 40°N, ranging from 0.2 to over 0.5 W m^–2^ (Fig. 5c). It can be due to more vigorous evaporation of water vapour by melting sea ice north of 50°N. The difference in latent heat change of more than 1 W m^–2^ between 70 and 80°N was consistent with the location of the large difference in surface albedo associated with sea ice melt (Fig. S1a). The zonal mean difference in the surface air temperature change was also large between 40 and 50°N where the difference in the surface radiation budget change was small (Fig. 5b). Therefore, the difference in the latent heat change can be a primary factor in this latitude due to a large difference in the column water vapour change over North Atlantic and East Asia (Fig. S1c). Although there are differences in cloud changes as sulfate aerosols decrease under different CO_2_ concentrations (Fig. S1d–S1e), the main changes are due to a difference in the latitude of the Intertropical Convergence Zone (Fig. S3) and are not expected to make the difference in the global temperature change.

Some studies have shown that analysis of the multi-model results of CMIP Phase 5 (CMIP5) indicates that future anthropogenic warming may alter climate sensitivity in general, with various feedbacks, such as cloud, water vapour, and ice-albedo feedbakcs, occurring on different time scales^19,20^. The results of this study indicate an irreversible change in surface air temperature with decreasing SO_2_ emissions under increasing CO_2_ concentrations with changing in the climate sensitivity, although the radiative forcing returns to the preindustrial level. As shown in Fig. 3a, zero SO_2_ emissions from fuel sources are expected to cause an increase in the global mean surface air temperature of 0.35 °C under a doubled CO_2_ concentration. The increase in temperature with decreasing sulphate aerosols is more spatially heterogeneous than that for well-mixed greenhouse gases, which is ≥ 1 °C over land in the Northern Hemisphere and in the cryosphere (Fig. 4). The Paris Agreement discussed controlling global mean surface air temperature increases to < 2 °C relative to preindustrial conditions and identified efforts aimed at limiting this increase to 1.5 °C. Local climate change due to mandatory reductions in SO_2_ emissions and the resulting decreases in sulphate aerosol concentrations to mitigate health impacts will be analysed in greater detail in future studies, together with other SLCFs including methane, tropospheric ozone, organic aerosols, and hydrofluorocarbons. In parallel with quantitative elucidation of the influence of SLCFs, synthesised mitigation measures for SLCFs should be promoted by established organisations, e.g., the Climate and Clean Air Coalition (CCAC) and Asia Pacific Clean Air Partnership (APCAP).

## Methods

In this study, a global coupled atmosphere–ocean general circulation model, MIROC^10^, was used, which calculates the time-integrated surface pressure and three-dimensional wind, temperature, specific humidity, and mass mixing ratios of liquid water and ice crystals. MIROC was coupled with the SPRINTARS aerosol module, which predicts the mass mixing ratios of major aerosol components (sulphate, BC, organic matter (OM), soil dust, sea salt, and precursor gases of sulphate, including SO_2_ and dimethyl sulphide (DMS)) by simulating the aerosol transport processes of emission, advection, diffusion, chemical reactions of sulphur, wet deposition, and dry deposition. SPRINTARS also simulated aerosol–radiation and aerosol–cloud interactions by connecting atmospheric radiation and cloud/precipitation processes, respectively, with MIROC. Detailed model descriptions of MIROC–SPRINTARS have been published previously^21^.

In this study, atmosphere-only and coupled ocean experiments were conducted using perturbed SO_2_ emissions from fuel sources with MIROC–SPRINTARS. In the former experiments, prescribed monthly mean data of sea surface temperature and sea ice from HadISST data in 2000^22^ were used to estimate instantaneous and effective radiative forcing. Instantaneous radiative forcing was calculated as the difference in the radiation budget due to aerosol–radiation interactions between two experiments with standard SO_2_ emissions and various scaling factors, such that changes in the radiation budget were calculated during the atmospheric radiation process with and without aerosols at each time step (i.e., double call). Due to the difference between two experiments, it could be affected by emission changes in natural aerosols, especially soil dust from dry regions (Sahara, Middle East, and southern Africa) (Fig. 1a). Effective radiative forcing was calculated as the simple difference in the radiation budget between the two experiments, including changes due to aerosol–radiation and aerosol–cloud interactions, with rapid adjustments to the meteorological field. It could change with perturbations of cloud and water vapour concentration by rapid adjustments (Fig. 1b). Coupled ocean experiments were performed to calculate changes in meteorological conditions (e.g., temperature, water vapour, and clouds), including all interactions and feedbacks. Simulations were integrated for 15 and 100 years in the atmosphere-only and coupled ocean experiments, respectively, and the simulated results were analysed for the last 10 and 50 years, respectively. The horizontal resolution was T85 (approximately 1.4° × 1.4° in longitude and latitude), and the vertical resolution of the hybrid sigma-pressure coordinate was 40 layers. The Emissions Database for Global Atmospheric Research and Task Force on Hemispheric Transport of Air Pollution (EDGAR–HTAP)^23^ and Global Fire Emissions Database (GFED)^24^ were used as emission inventories for fuel and biomass burning sources, respectively, of SO_2_, BC, and OM. Natural SO_2_ emissions from volcanoes^23^ and DMS from oceanic phytoplankton and land vegetation were also included in the simulation.

## Supplementary Information

Supplementary Figures.
