# Supplementary material for: Return to different climate states by reducing sulphate aerosols under future CO2 concentrations
Source: Sci Rep. 2020 Dec 10;10:21748. doi: 10.1038/s41598-020-78805-1 (PMC7729963; doi:10.1038/s41598-020-78805-1)
Supplement: Supplementary file 1 — Supplementary Figures. [file 41598_2020_78805_MOESM1_ESM.docx]

**Supplementary information:**

**Return to different climate states by reducing sulphate aerosols under future CO_2_ concentrations**

*Toshihiko Takemura^1^

^1^Research Institute for Applied Mechanics, Kyushu University, Fukuoka, Japan

*Corresponding author: Toshihiko Takemura (toshi@riam.kyushu-u.ac.jp)

**a b**


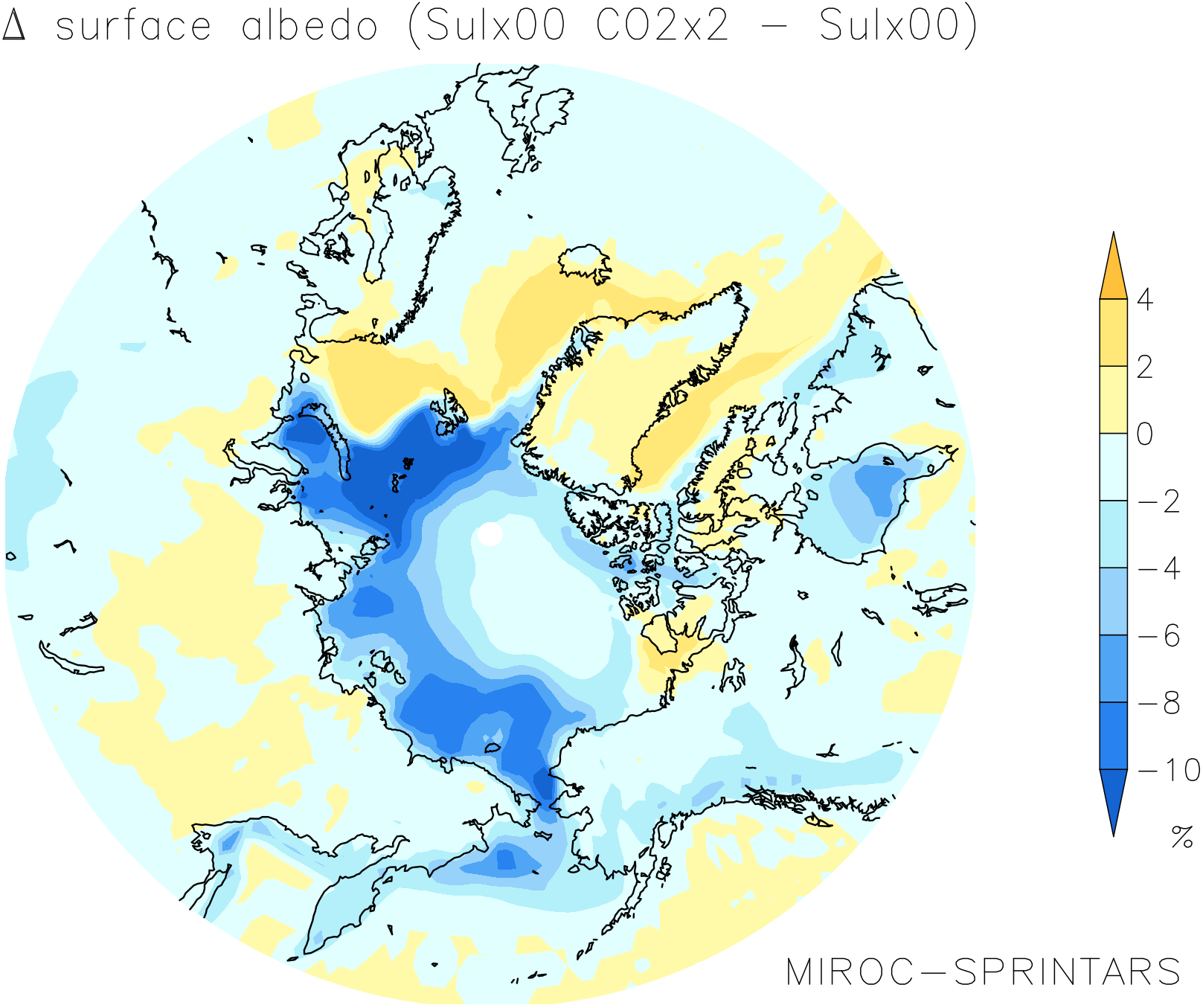
 　
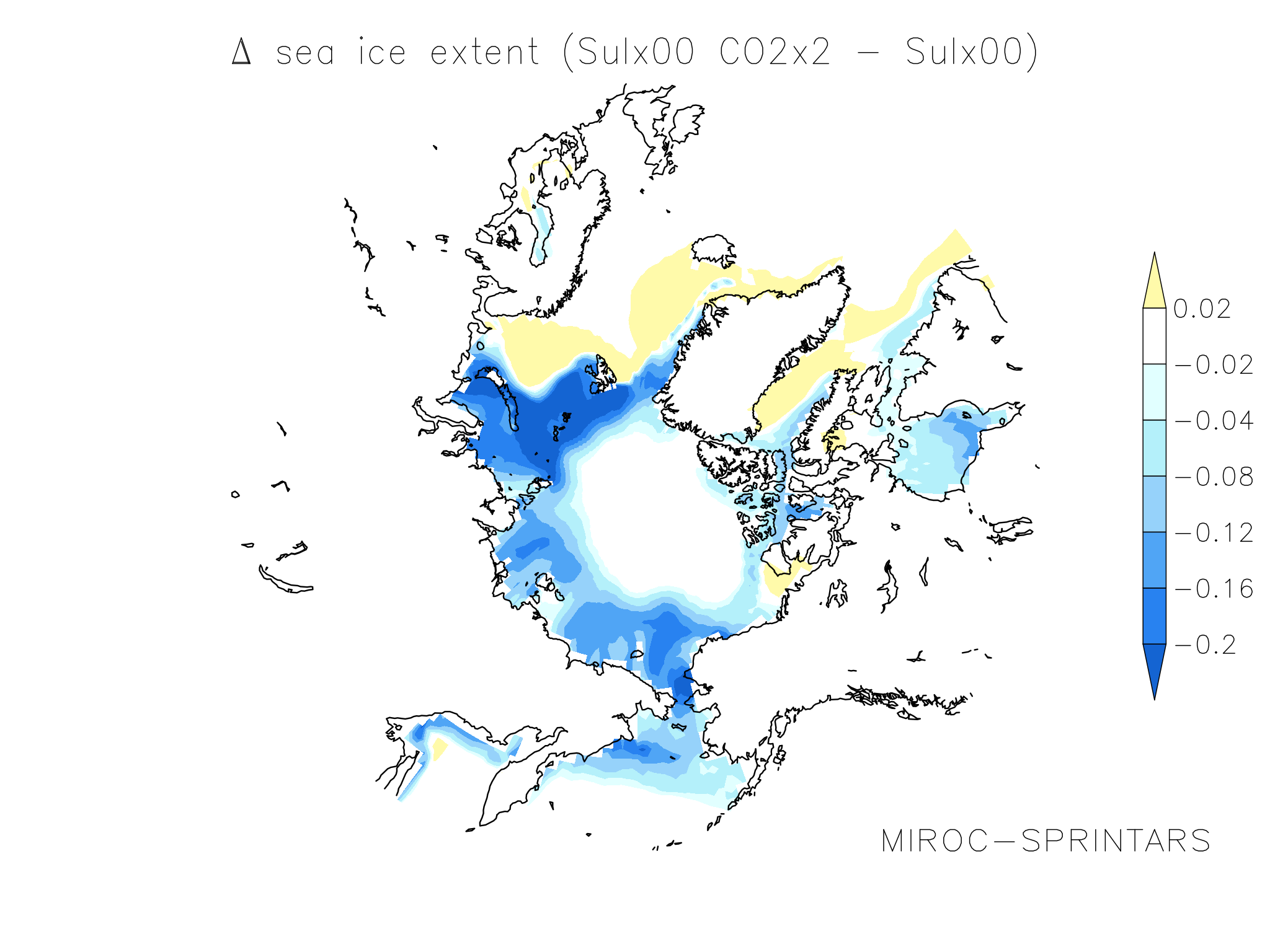


**c d**


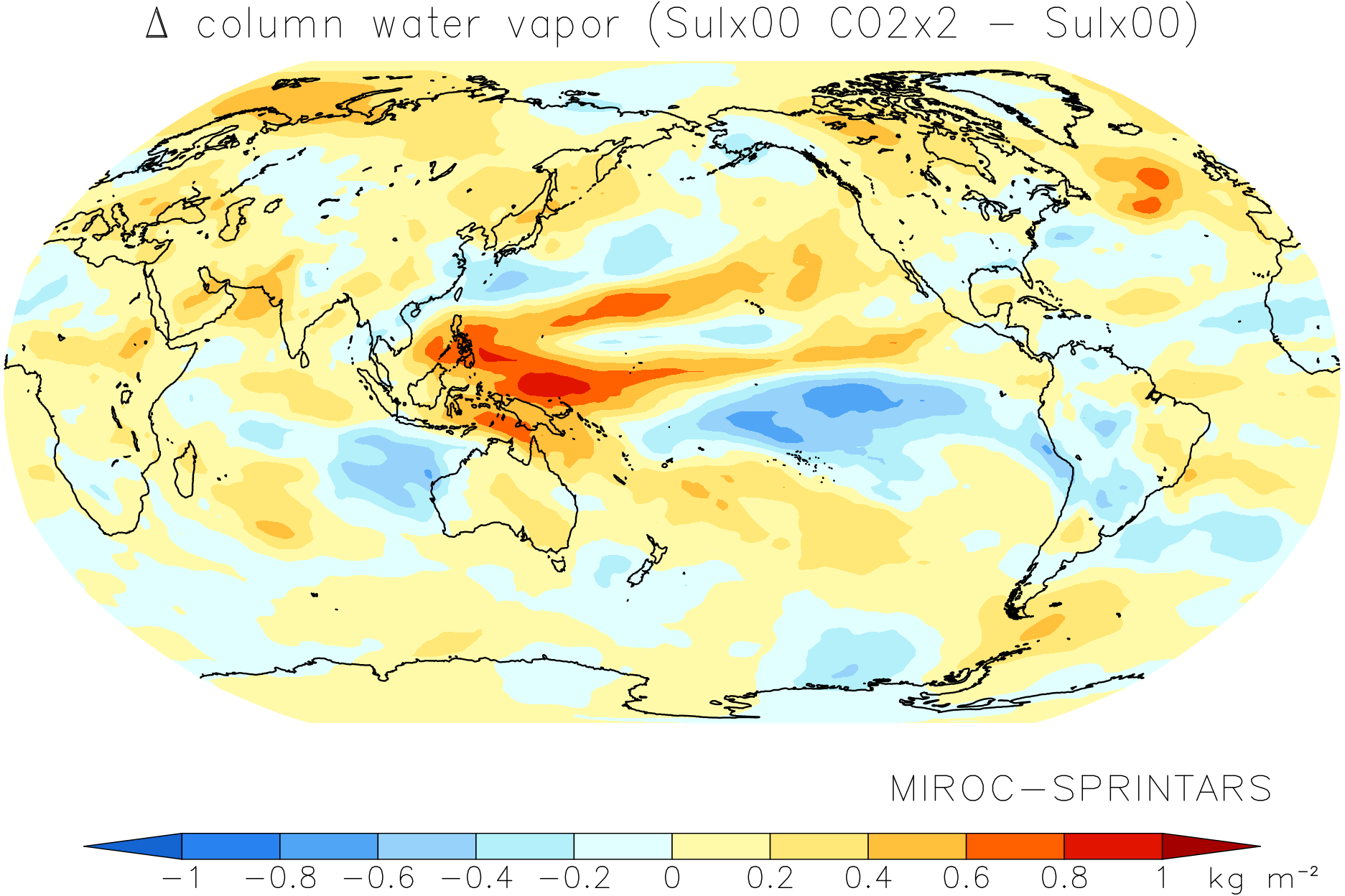

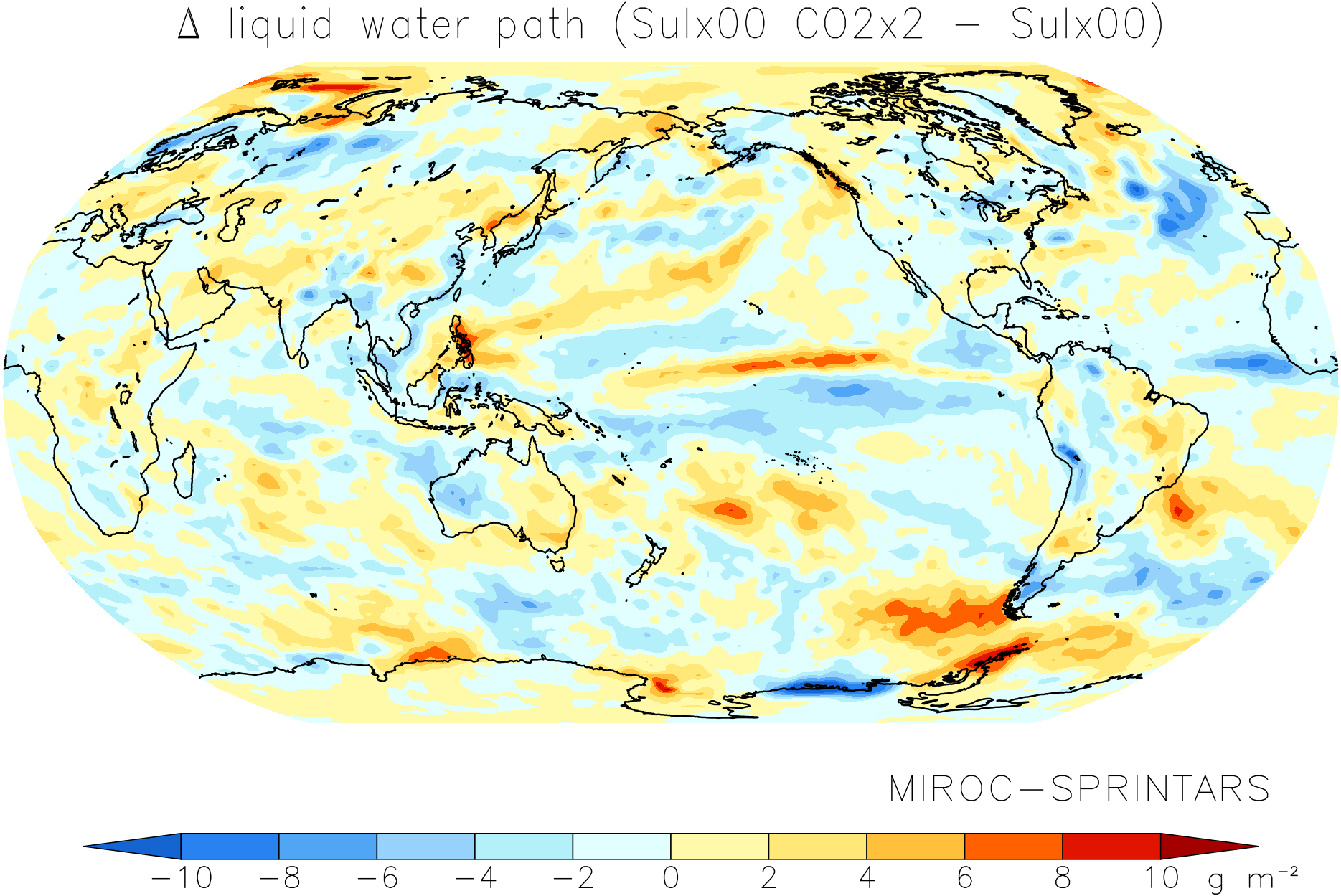


**e**


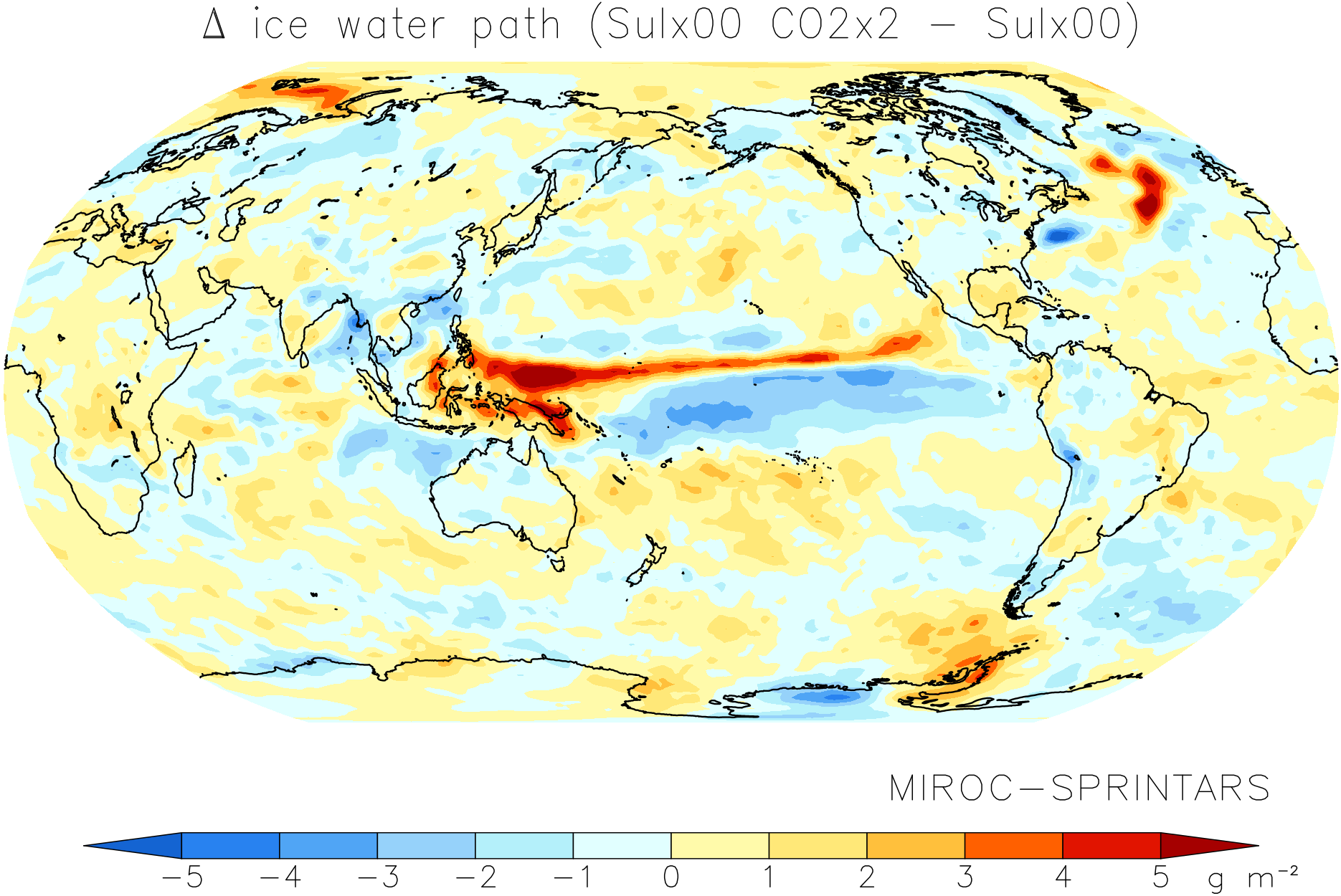


**Supplementary Figure S1 | Changes in parameters due to reducing SO_2_ emissions to zero under different CO_2_ concentrations.** Distributions of annual mean differences in the change in the surface albedo at the shortwave radiation (**a**), sea ice extent (**b**), column water vapor (**c**), liquid water path (**d**), and ice water path (**e**) following reduction of SO_2_ emissions from fuel sources to zero with doubled CO_2_ concentration relative to the present. The sea ice extent is defined as 1 if the monthly mean sea ice concentration is more than 15%. The maps were generated with GrADS 2.2.1 (URL: http://cola.gmu.edu/grads/).

**a**
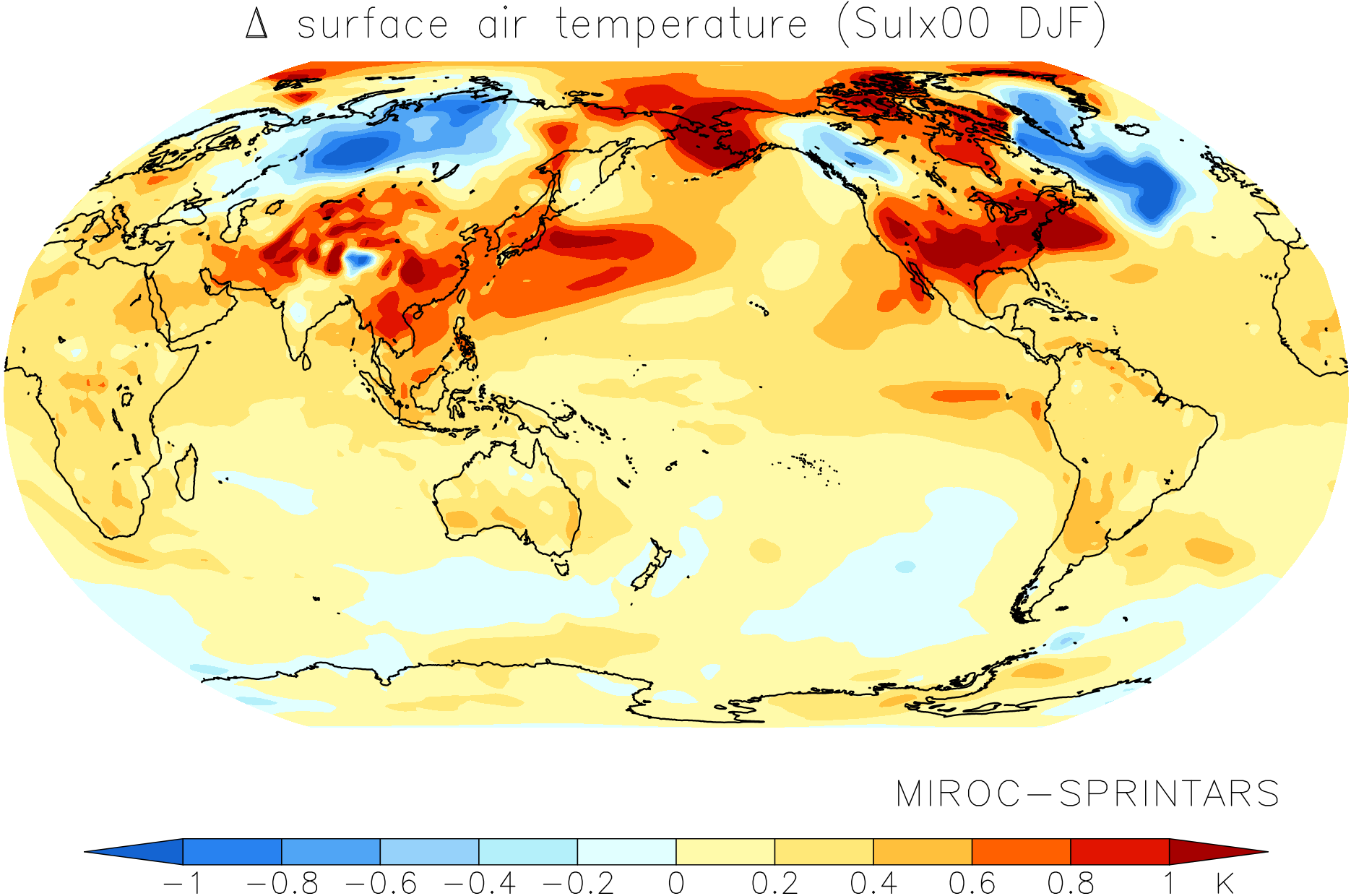

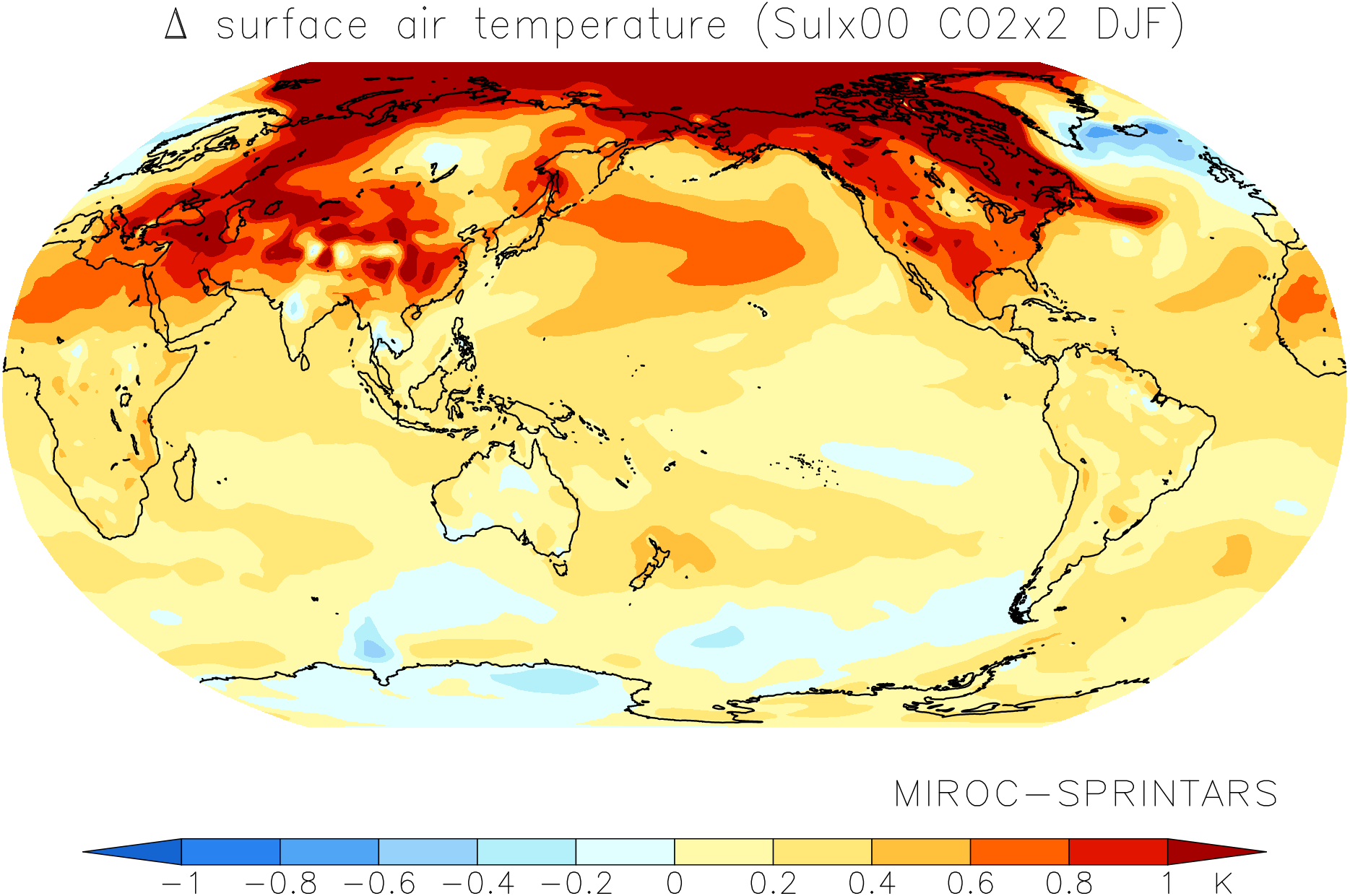


**b**
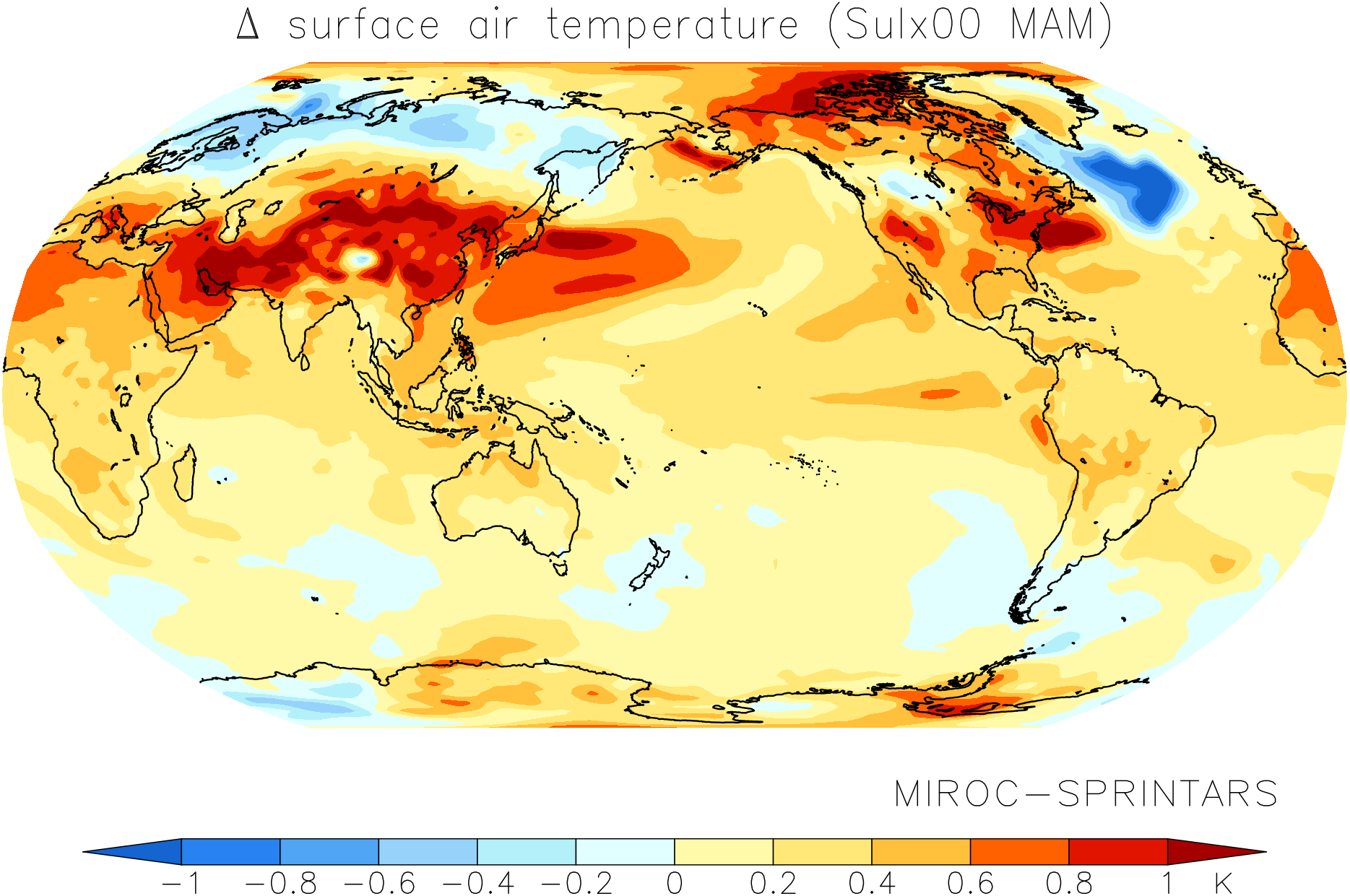

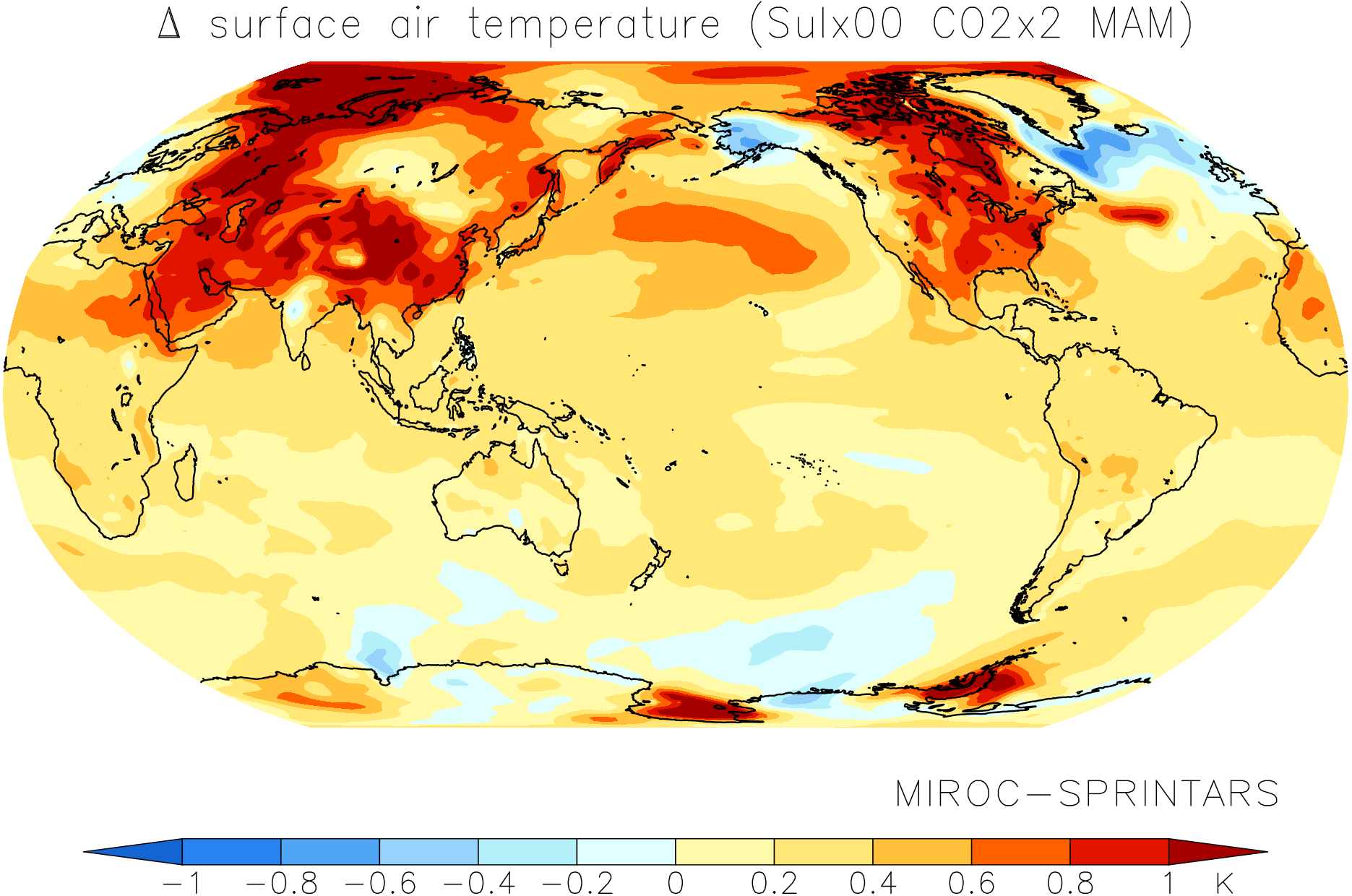


**c**
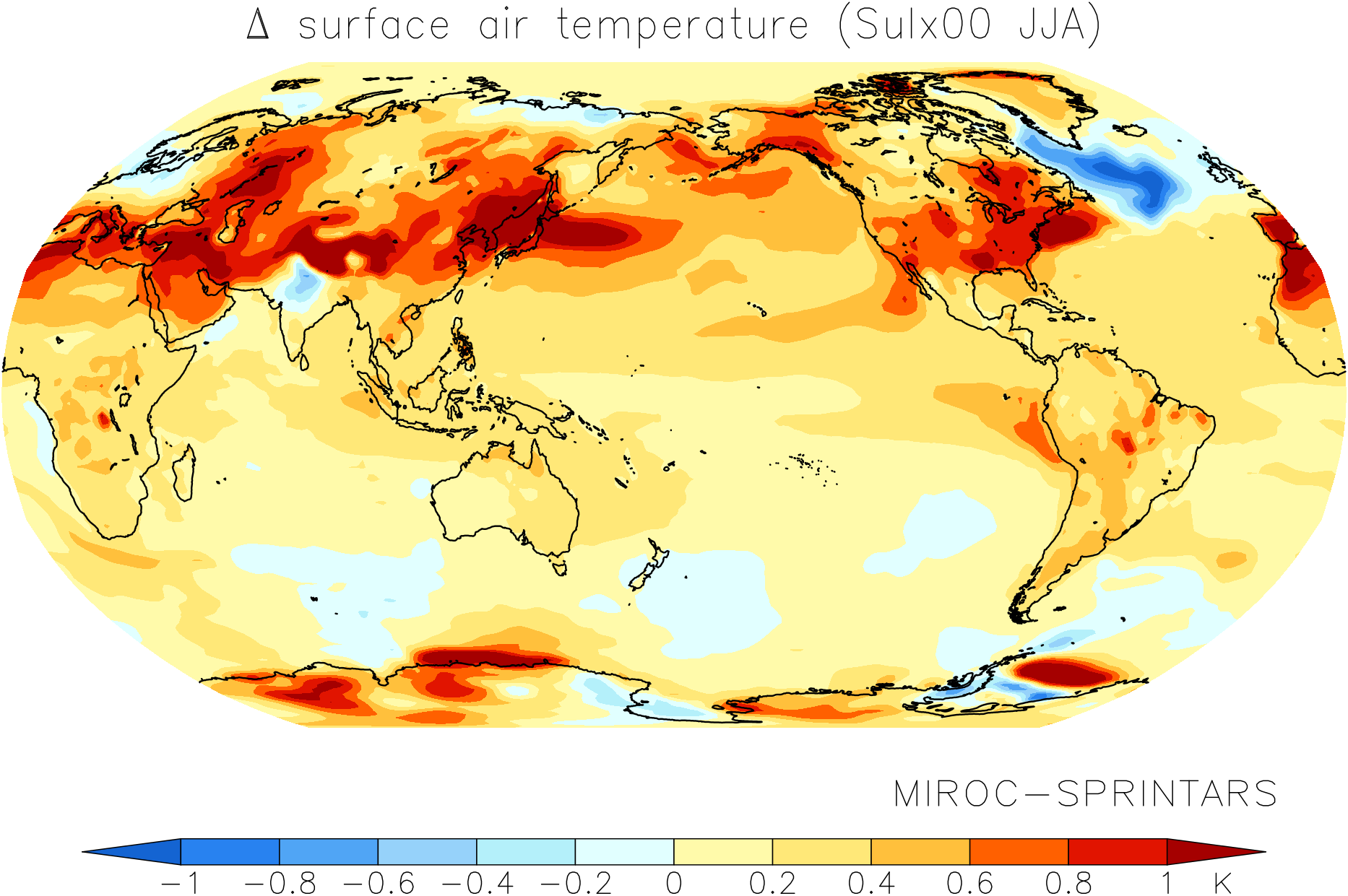

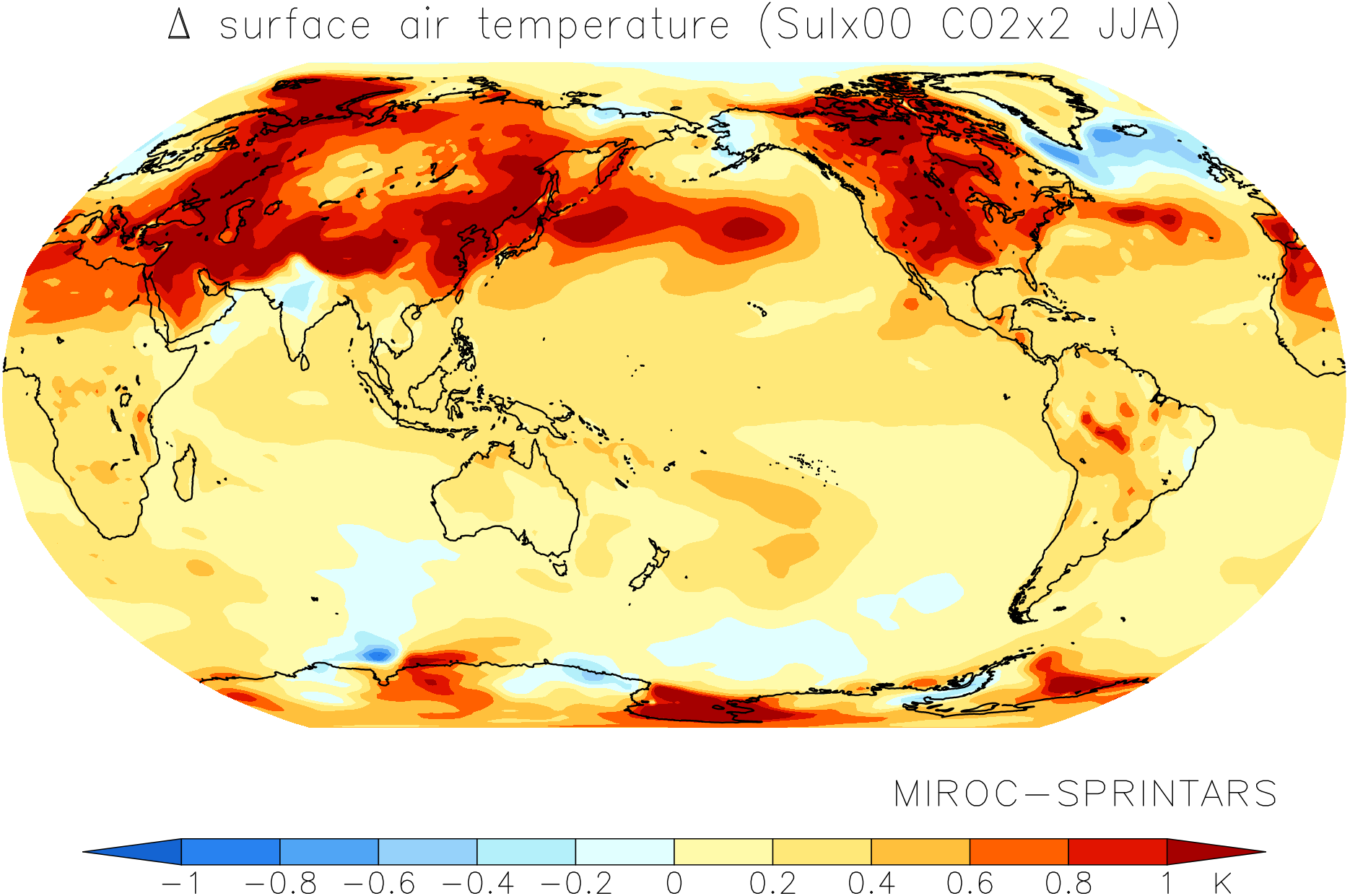


**d**
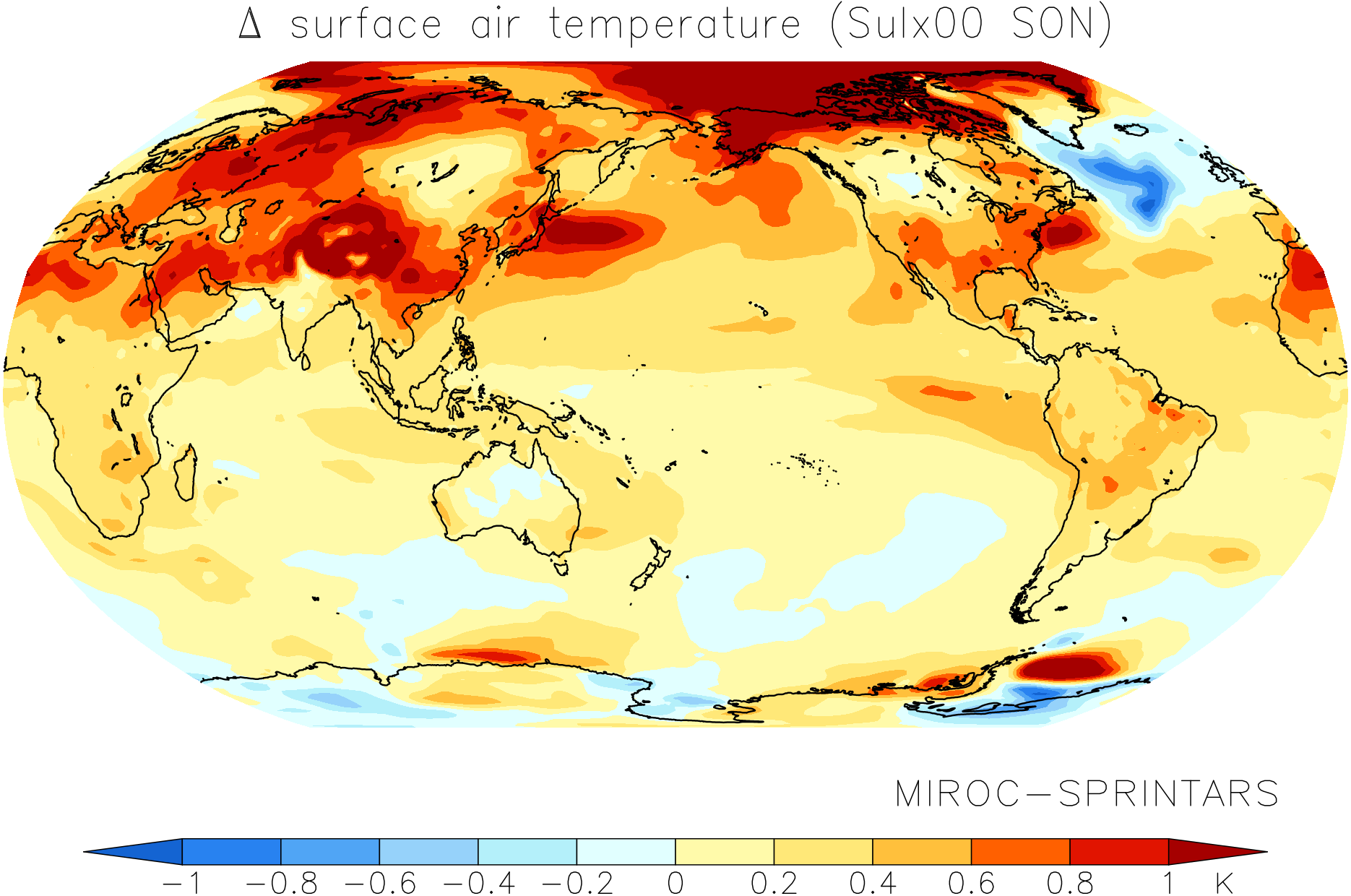

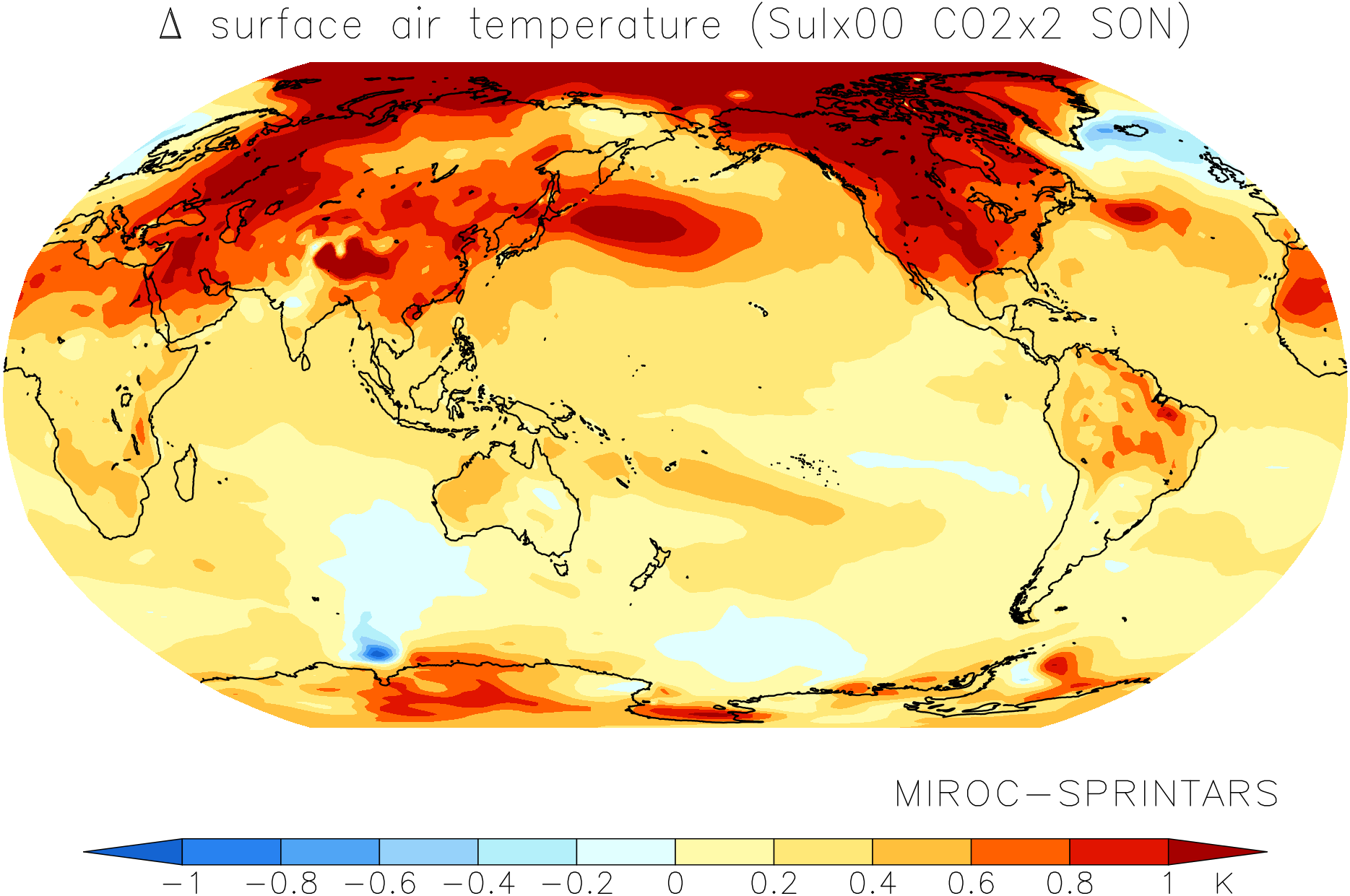


**Supplementary Figure S2 | Seasonal changes in surface air temperature due to reducing SO_2_ emissions to zero.** Seasonal mean distributions of changes in the surface air temperature following reduction of SO_2_ emissions from fuel sources to zero under present (left) and doubled (right) CO_2_ concentrations in boreal winter (**a**), spring (**b**), summer (**c**), and autumn (**d**). The maps were generated with GrADS 2.2.1 (URL: http://cola.gmu.edu/grads/).

**a b**


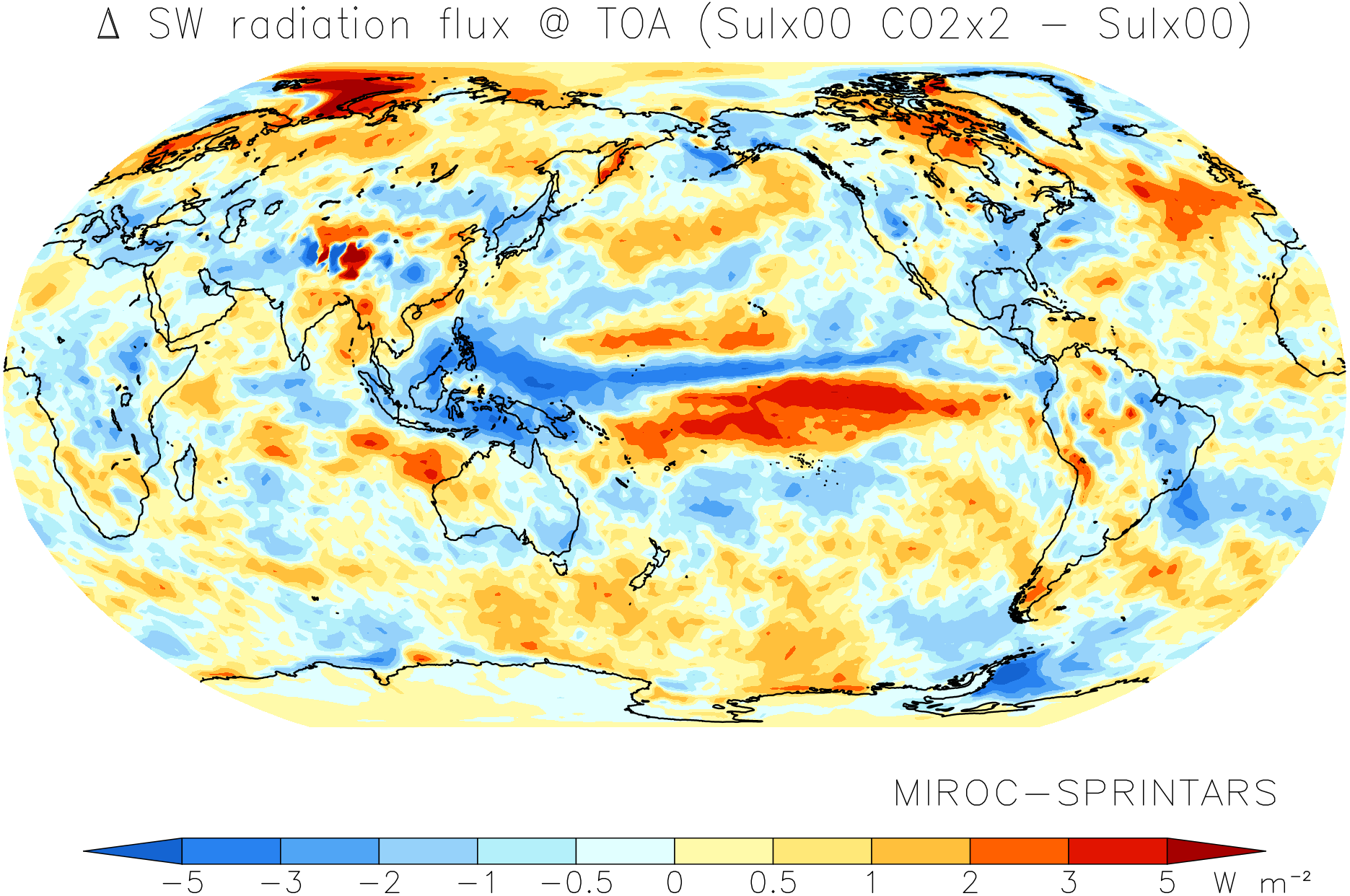

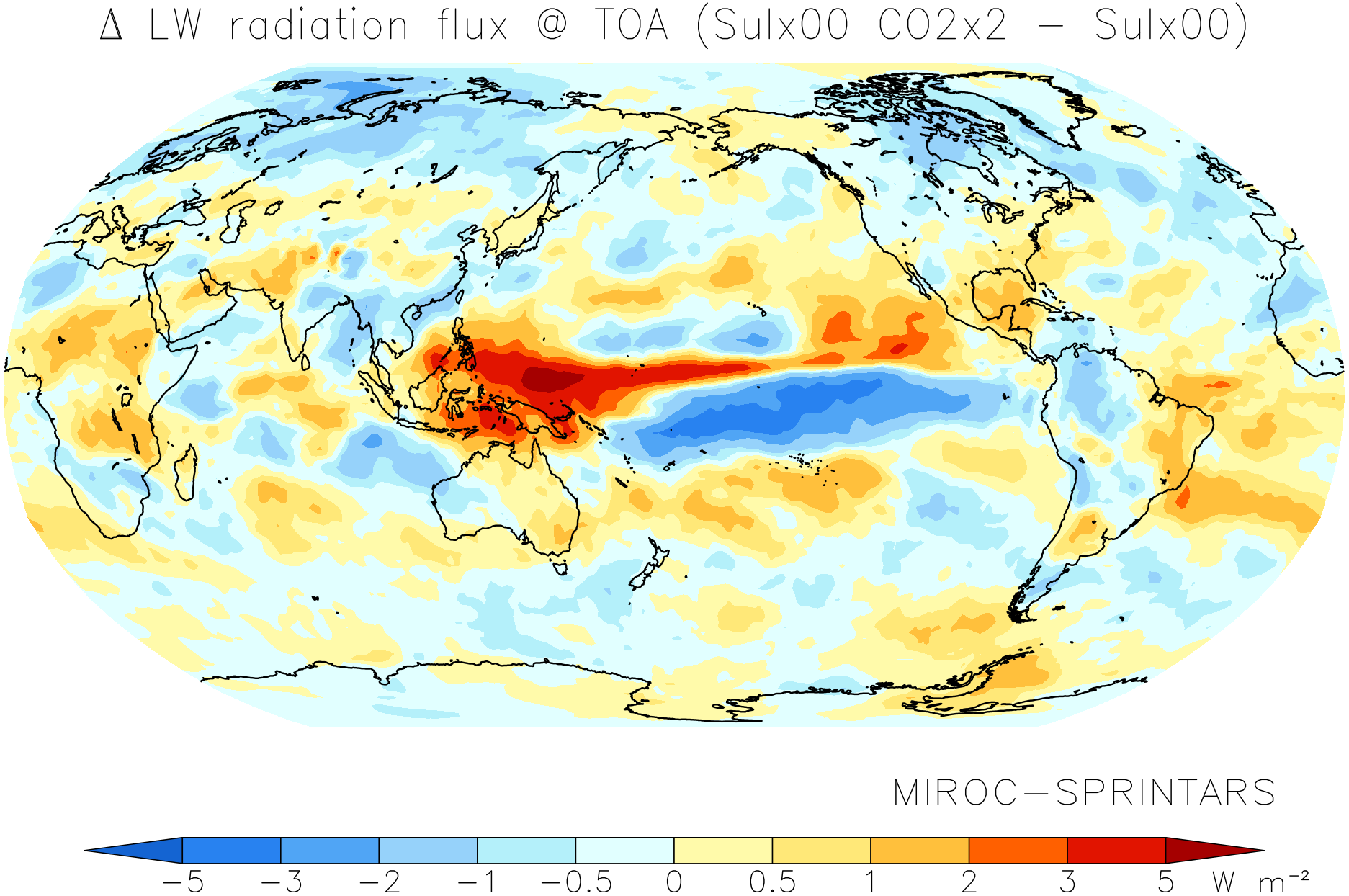


**c d**


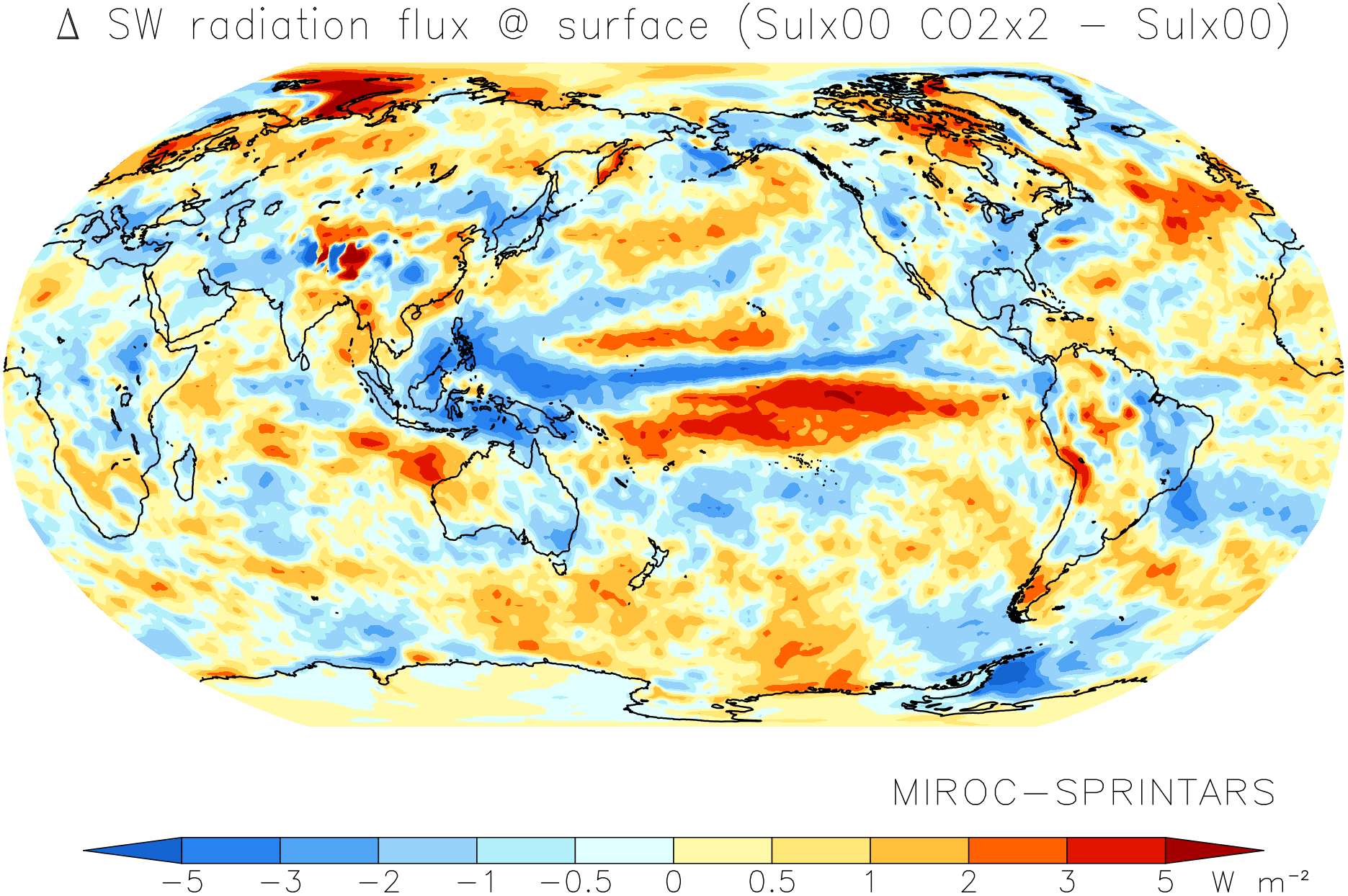

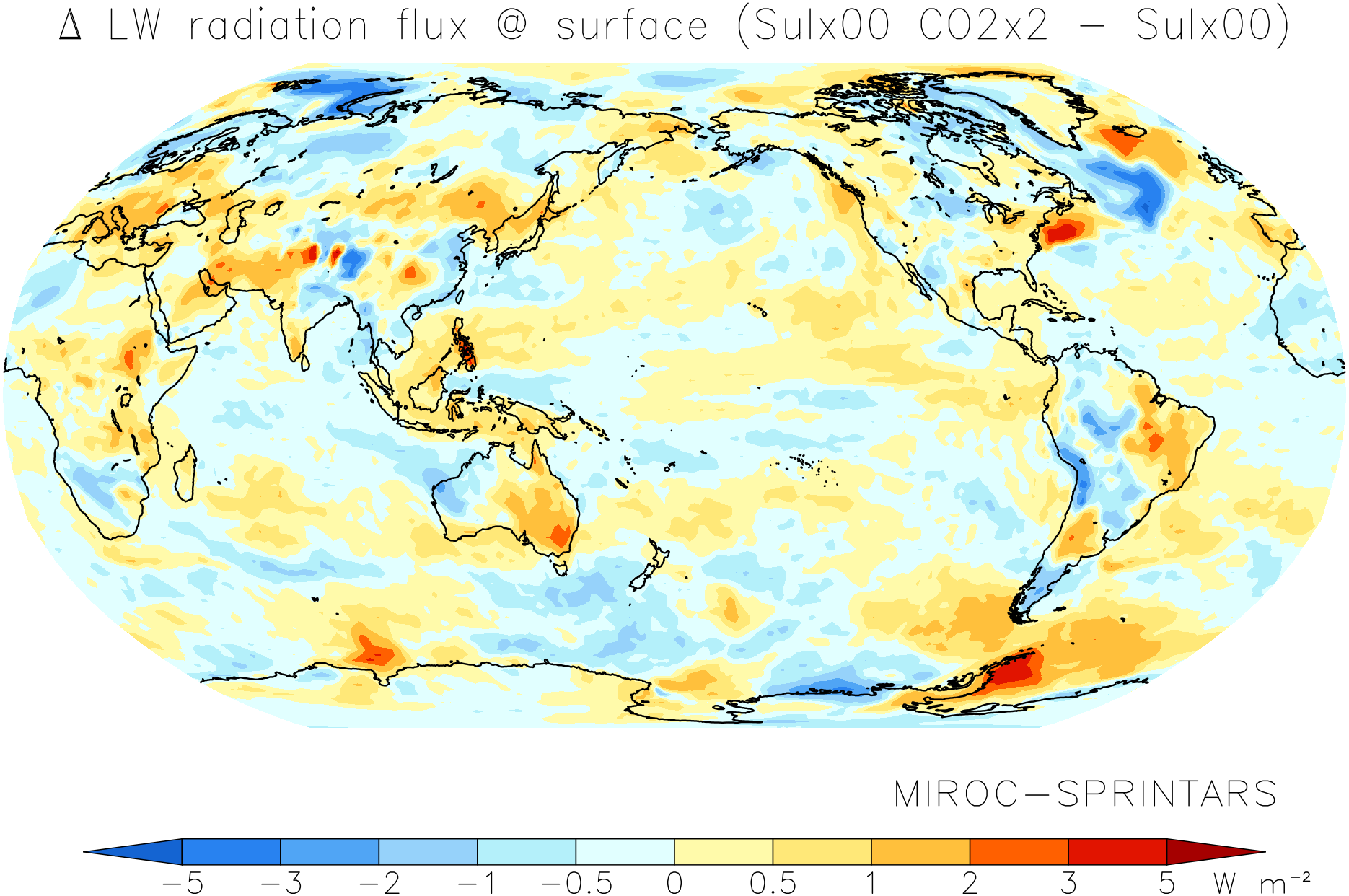


**Supplementary Figure S3 | Changes in radiation budget due to reducing SO_2_ emissions to zero under different CO_2_ concentrations.** Distributions of annual mean differences in the change in the radiation budget following reduction of SO_2_ emissions from fuel sources to zero with doubled CO_2_ concentration relative to the present for shortwave (**a**) and longwave (**b**) radiation at the top of the atmosphere and for shortwave (**c**) and longwave (**d**) radiation at the surface. The upward radiation flux is positive. The maps were generated with GrADS 2.2.1 (URL: http://cola.gmu.edu/grads/).
